# Body weight in systemic lupus erythematosus is associated with disease activity and the adaptive immune system, independent of type I IFN

**DOI:** 10.3389/fimmu.2025.1503559

**Published:** 2025-02-18

**Authors:** Hiroyuki Teruya, Hirofumi Shoda, Takahiro Itamiya, Yumi Tsuchida, Tomohisa Okamura, Keishi Fujio

**Affiliations:** ^1^ Department of Allergy and Rheumatology, Graduate School of Medicine, The University of Tokyo, Tokyo, Japan; ^2^ Department of Rheumatology, Tokyo Medical University Hospital, Tokyo, Japan; ^3^ Department of Functional Genomics and Immunological Diseases, Graduate School of Medicine, The University of Tokyo, Tokyo, Japan

**Keywords:** SLE, type I interferon, obese, cachexia, acquired immunity, transcriptome, immunophenotyping

## Abstract

**Objective:**

To explore the relationship between physique and immunological disturbances in systemic lupus erythematosus (SLE), we analyzed the clinical, immunological and transcriptomic characteristics of patients with SLE in relation to body mass index (BMI).

**Methods:**

Clinical characteristics were obtained from patient charts, and serum cytokine levels were measured. Phenotypes and transcriptomes of peripheral immune cells from patients with SLE in the ImmuNexUT database were analyzed in relation to BMI.

**Results:**

Thirty-four SLE patients were included in the analysis. Fever and mucocutaneous symptoms were commonly observed in SLE patients with a low BMI. BMI was negatively correlated with the SLE disease activity (SLEDAI)-2K scores. Multiple regression analysis revealed that BMI was an independent explanatory variable for SLEDAI-2K scores, irrespective of anti-dsDNA antibody or complement levels. Although serum interferon (IFN)-alpha and IFN-gamma levels were negatively associated with BMI, causal mediation analysis showed that BMI had a direct effect on SLEDAI-2K scores, independent of IFN-alpha levels. Immunophenotyping indicated that BMI was primarily correlated with T cell subsets. BMI-related gene expression was mainly enriched in the regulatory T cells and B cell subsets. BMI was negatively correlated with several cellular metabolic pathways, including glucose metabolism-related pathways in Th1 and effector memory CD8^+^ T cells, but not with IFN signaling.

**Conclusion:**

We characterized the clinical, immunological and transcriptomic profiles of SLE patients with varying BMI. As low BMI was identified as an independent parameter for explaining disease activity, cachexia is considered one of the systemic symptoms of active SLE. Additionally, BMI influenced the phenotypic and transcriptomic alterations of acquired immune cells, independent of IFN signaling. These findings provide insights into the pathogenesis of SLE.

## 1 Introduction

Systemic lupus erythematosus (SLE) is a systemic disorder that effects multiple organs and tissues, including nephritis, cutaneous lesions, serositis, and arthritis (1, 2). Autoimmune dysregulation plays a substantial role in the pathogenesis of SLE, particularly through the actions of immune cells and pro-inflammatory cytokines, which drive both organ-specific and systemic inflammation (1, 2). Among these cytokines, interferons (IFNs) are especially critical in the pathogenesis of SLE (3, 4). The IFN family comprises three types: type 1,2 and 3, each of which contributes to the pathogenesis of SLE. For instance, type 1 IFNs not only induce febrile reactions but also enhance autoimmune responses by activating autoantigen presentation on dendritic cells and promoting the differentiation of autoreactive B cell clones (5). Notably, anifrolumab, an anti-IFN RA monoclonal antibody, blocks type 1 IFN signaling and has shown efficacy in controlling disease activity in SLE (6, 7). Additionally, various immune cells, including B cells, T cells, monocytes, and neutrophils, also contribute to the pathogenesis of SLE (1, 2). In this way, immune dysregulation plays a pivotal role in the pathogenesis of SLE; however, the detailed mechanisms remain unclear.

Recently, the relationship between the physique and the immune system has become a focus of research, particularly in the context of obesity and immune dysregulation (8). For example, adipokines, secreted by adipocytes and muscle cells, can influence the immune system (9). In particular, leptin, secreted from adipocytes, not only regulates appetite, but also activates both innate and acquired immune cells, promoting inflammatory responses (10). In addition, increasing evidence suggests that obesity induces low-grade inflammation and alters immune cell functions (8, 11). For example, a recent study demonstrated that obesity had a promoting effect on Th2 and Th17-related diseases, such as atopic dermatitis (12). In regards of COVID-19 infection, obesity has been identified as a potent risk factor for severe disease (13). Several explanations for the role of obesity in disease have been proposed, and many researchers suggested that the activation of M1 macrophages and the excessive release of pro-inflammatory cytokines, such as IL-6 and TNF-alpha, are elevated in obese patients (14). In the field of autoimmune diseases, obesity is considered one of the major contributors to psoriasis (15). We have also reported Th17 cell activation and elevated IL-1 levels in obese rheumatoid arthritis patients (16). The relationship between physique and type 1 IFNs, which are key mediators of SLE pathogenesis and disease activity, is contradictory. For example, IFN-alpha promotes adipocyte-intrinsic inflammatory potential in the context of insulin resistance (17). On the other hand, some studies have found that type 1 IFN production is reduced in non-obese individuals compared to those with obesity (18). Certain researchers suggested that leptin suppresses type 1 IFN-related responses in obesity (19). Additionally, clinical observations examining the association between physique and disease activity in SLE patients have yielded mixed results (20–22). In the Hopkins Lupus Cohort, more than half of SLE patients exhibited cachexia, which was associated with various lupus manifestations and a higher damage index (20). Conversely, smaller studies have indicated that obesity may increase the risk of disease deterioration and flare-ups (21, 22). Thus, the relationship between physique and SLE remains controversial, and further investigation of physique-related immunological characteristics is required to clarify this connection.

We established a transcriptomic catalog of immune cells in autoimmune diseases, the Immune Cell Gene Expression Atlas from the University of Tokyo (ImmuNexUT) (23). The ImmuNexUT database allows for transcriptome analysis in autoimmune diseases, including SLE (24). This study aimed to analyze the transcriptomic and immunological characteristics of patients with SLE in relation to body mass index (BMI) using the ImmuNexUT database, with the goal of advancing our understanding of SLE pathogenesis and facilitating clinical applications, such as personalized medicine.

## 2 Materials and methods

### 2.1 Participants

Patients with new-onset or flare-up SLE, who were hospitalized at the University of Tokyo Hospital between September 2017 and June 2020, were enrolled in this study. All patients fulfilled the Systemic Lupus International Collaborating Clinics (SLICC) 2012 and the American College of Rheumatology (ACR) 2019 classification criteria for SLE (25, 26). Clinical and demographic information, such as BMI, laboratory data and SLE Disease Activity Index 2000 (SLEDAI-2K) scores (27) and their components were extracted from medical records. Written informed consent were obtained from all participants. This study was approved by our local ethics committees (11592, G10095) and was performed in accordance with the latest version of the Declaration of Helsinki.

### 2.2 ImmuNexUT

Transcriptome data of the participants were obtained from the ImmuNexUT database (23). Data of SLE patients, whose BMI data could be obtained, were utilized for transcriptomic analysis. Detailed information on ImmuNexUT has been previously described (23). RNA sequencing data were analyzed using the R version 4.3.3 (R Foundation for Statistical Computing). Using the MASS package, we calculated the genes related to BMI by regressing the expression levels of each subset of genes with BMI, age, daily dosage of prednisolone (PSL), and library size as explanatory variables. A false discovery rate (FDR) of < 0.01 was regarded as significant. Pathway analysis was performed using the ReactomePA package (28), and cell metabolism pathways were referenced from scMetabolism (29). Gene set variation analysis (GSVA) was performed to calculate the signature scores for these pathways (30).

### 2.3 Immunophenotyping

Immunophenotyping of peripheral blood cells was performed using a FACS Aria (BD Biosciences). The 24 immune cell subsets analyzed were as follows: naïve CD4^+^ T cells (naïve CD4), memory CD4^+^ T cells (Mem CD4), Th1 cells, Th2 cells, Th17 cells, T follicular helper cells (Tfh), fraction I naïve Tregs (Fr. I nTregs), fraction II effector Tregs (Fr. II eTregs), fraction III non-regulatory T cells (Fr. III T), naïve CD8^+^ T cells (naïve CD8), central memory CD8^+^ T cells (CM CD8), effector memory CD8^+^ T cells (EM CD8), NK cells, naïve B cells (naïve B), unswitched memory B cells (USM B), switched memory B cells (SM B), double negative B cells (DN B), plasmablasts (plasmablast), classical monocytes (CL Mono), intermediate monocytes (Int Mono), non-classical monocytes (NC Mono), myeloid dendritic cells (mDC), and plasmacytoid dendritic cells (pDC). The proportion of each subset was calculated. Detailed definitions of these subsets are provided in our previous article (31).

### 2.4 Measurement of serum cytokines and adipokines

Serum cytokines and functional proteins were measured using multiplex ELISA, Luminex assays (R&D Systems). Serum leptin and ghrelin levels were measured using ELISA (R&D systems).

### 2.5 Statistics

R (ver.4-3-3) was used for all statistical analyses. Differences between two groups of non-normally distributed continuous data were tested for significance with nonparametric Mann-Whitney U test or Fisher’s exact test. The BMI cutoff value was set at 20 in accordance with the previous study (32). Correlations were evaluated by nonparametric Spearman’s rank correlation coefficients. The contribution to the disease activities was analyzed by multiple regression analysis. Causal mediation analysis was explored by Package “Mediation” (33) and *p*-values were calculated via 1000-time bootstrapping. P values < 0.05 were considered significant.

## 3 Results

### 3.1 Characteristics of patients with SLE

Thirty-four patients with SLE were enrolled in the study, and their demographic and clinical information are summarized in **Supplementary Table 1**. Eighty-two percent of the patients were female with a mean age was 48.6 ± 14.6 years old. The mean SLEDAI-2K score was 11.8 ± 7.6. Fifty-two percent of the patients were taking glucocorticoid, with a mean daily doses of 6.3 ± 11.9. The mean BMI was 21.6 ± 2.9.

### 3.2 Association between disease activity and BMI in patients with SLE

**Figure 1:**
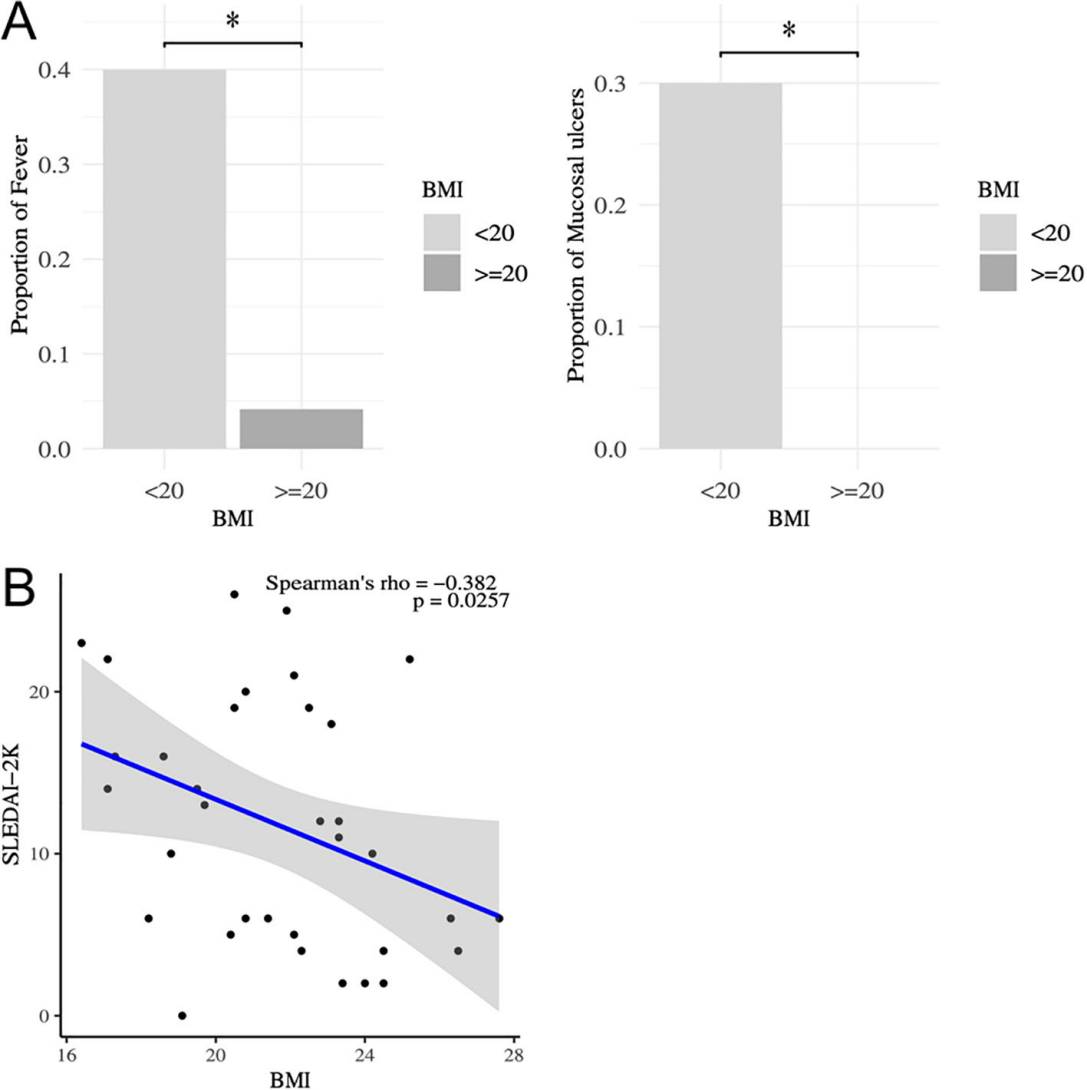
Correlation between SLEDAI-2K and BMI in patients with SLE. **(A)** Comparison of the frequencies of SLE symptoms between those with low BMI (BMI<20) and others by Fisher’ exact test. *P < 0.05. **(B)** Correlation between SLEDAI-2K and BMI analyzed by Spearman’s rank correlation coefficients in patients with SLE (n=34). Comparison of SLEDAI-2K between those with low BMI (BMI<20) and others by Mann-Whitney U-test. *P < 0.05. SLEDAI, systemic lupus erythematosus disease activity index; BMI, body mass index.

First, the association between BMI and clinical parameters was analyzed in patients with SLE (**Table 1**). Fever and mucocutaneous symptoms were negatively correlated with BMI, and these symptoms were significantly more frequent in SLE patients with a lower BMI (**Figure 1A**). Importantly, SLEDAI-2K scores were significantly correlated with BMI (**Figure 1B**). Several previous studies and clinical experiences support the idea that serum anti-dsDNA antibody titers and complement levels are weighted and reliable indicators of disease activities in SLE (27, 34). Therefore, we examined the additional effect of BMI on these serum indicators to predict the SLEDAI-2K scores using multiple regression analysis (**Table 2**). When we performed multiple regression analysis on serum anti-dsDNA antibody titer and complement (CH50) levels (model 1), the coefficient of determination was 0.37 (*p* = 0.00037). Importantly, when we added BMI to these parameters (model 2), there was still a significant difference in BMI, and the coefficient of determination was 0.43 (*p* = 0.00018). We tested the significance of BMI by comparing two models using an analysis of variance. The inclusion of BMI in the model significantly improved the prediction of the SLEDAI-2K scores (*p* = 0.03987). Thus, it was demonstrated that low BMI was independently associated with disease activity in patients with active SLE, especially in those with fever and mucocutaneous symptoms.

**Table 1:** The correlation between BMI and SLEDAI2K items.

Variable	Spearman Correlation	P-value
Psychosis	0.12426243	0.483810241
Visual Disturbance	0.07198362	0.685801866
Cranial Nerve Disorder	0.12426243	0.483810241
Lupus headache	-0.07010863	0.693585020
Vasculitis	-0.23964898	0.172220788
Arthritis	-0.32908913	0.057374669
Myositis	0.20620155	0.241999303
Urinary casts	-0.19035556	0.280884303
Hematuria	-0.13549859	0.444827235
Proteinuria	-0.20505252	0.244690926
Pyuria	-0.11856126	0.504243306
New Rash	-0.09834542	0.580028993
Alopecia	-0.12567299	0.478821707
Mucosal ulcers	-0.45470085	0.006898549**
Pleurisy	-0.22029375	0.210597699
Pericarditis	0.19083676	0.279647235
Low complement	-0.20395238	0.247286713
Increased anti-DNA antibodies	-0.33349976	0.053920937
Fever	-0.49965104	0.002624208**
Thrombocytopenia	-0.12567299	0.478821707
Leukopenia	-0.31111417	0.073296380

Correlations between SLEDAI-2K components and BMI analyzed by Spearman’s rank correlation coefficients in patients with SLE (n=34). **P <0.01. SLEDAI, systemic lupus erythematosus disease activity index; BMI, body mass index; CRP, C reactive protein; ESR, erythrocyte sedimentation rate; ds-DNA Ab, double-stranded DNA antibody.

**Table 2:** The multiple regression analysis.

Indicators		Estimate	Std. Error	t-value^1^	p^2^	Adjusted R^2^	F^3^	p^4^
Model 1	(Intercept)	13.32196	2.61481	5.095	1.78e-05***	0.3697	10.38	0.0003742
	CH50	-0.11661	0.06630	-1.759	0.08881			
	dsDNA	0.03350	0.01161	2.886	0.00717*			
Model 2	(Intercept)	30.20764	8.22667	3.672	0.000967***	0.4377	9.303	0.0001811
	CH50	-0.11928	0.06263	-1.904	0.066810			
	dsDNA	0.03035	0.01106	2.743	0.010318*			
	BMI	-0.76981	0.35775	-2.152	0.039873*			

Multiple regression analysis for the SLEDAI-2K scores. *P < 0.05, ***P < 0.001. SLEDAI, systemic lupus erythematosus disease activity index; BMI, body mass index; ds-DNA Ab, double-stranded DNA antibody; CH50, 50% hemolytic unit of complement. ^1^Coefficient t-value. ^2^Calculated probabilities of each independent variable. ^3^Test statistic from the F-test. ^4^Calculated probabilities of each model.

### 3.3 Association between serum cytokines and BMI in patients with SLE

**Figure 2:**
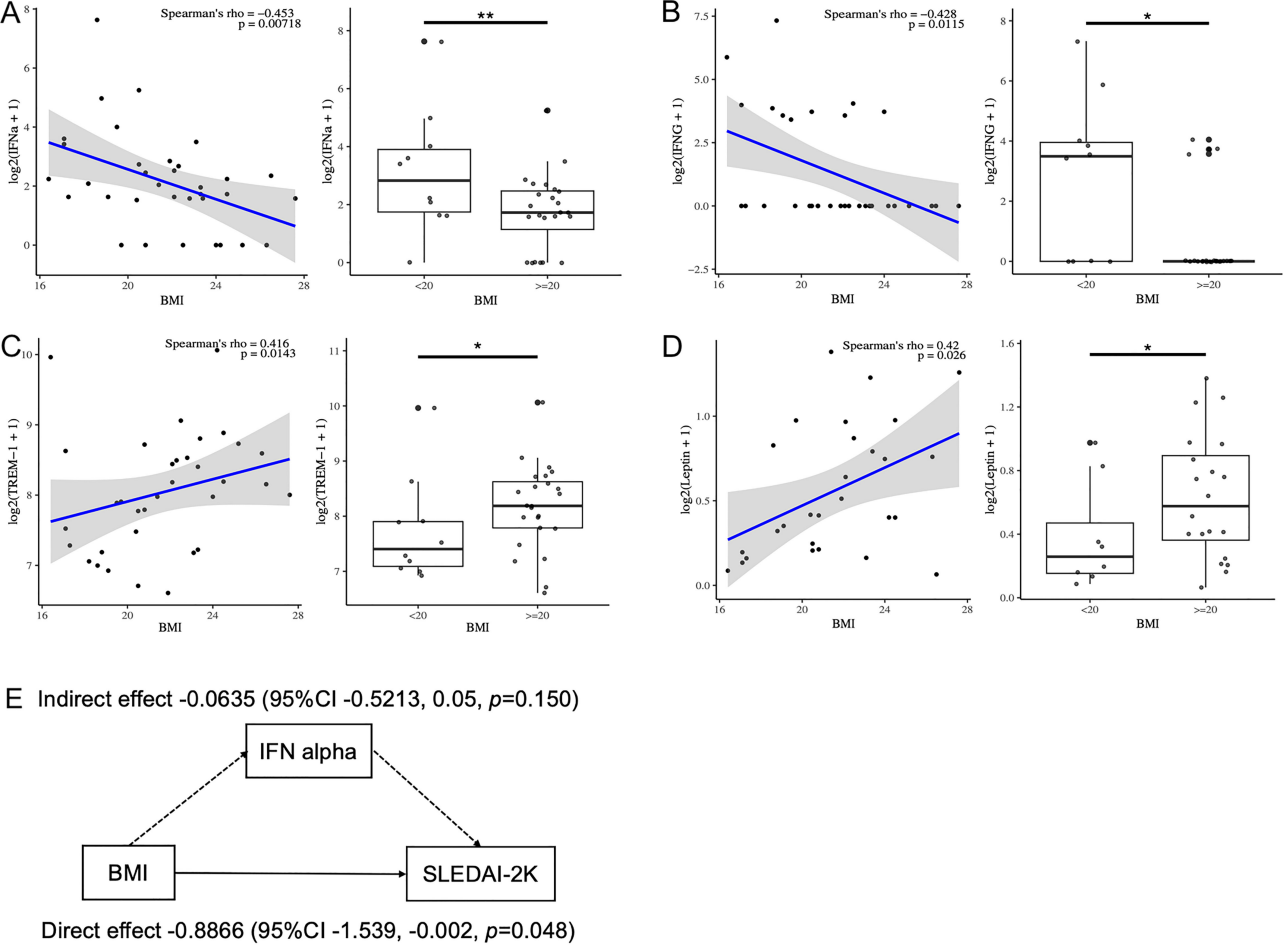
Correlation between serum cytokine levels and BMI in patients with SLE. **(A–D)** Correlation between serum IFN- a, IFN-g, leptin and TREM-1 levels and BMI analyzed by Spearman’s rank correlation coefficients in patients with SLE (n=34). Comparison of serum cytokine levels between those with low BMI (BMI<20) and others by Mann-Whitney U-test. **(E)** Diagram showing the results of causal mediation analysis. *P < 0.05, **P <0.01. BMI, body mass index; IFN, interferon; TREM, triggering receptor expressed on myeloid cells.

Next, to elucidate the association between serum cytokines and BMI in patients with SLE, serum cytokines and adipokines, including leptin and ghrelin, were measured (**Table 3**). Serum IFN-alpha, IFN-gamma, and IL-12/23p40 levels were negatively correlated with BMI, whereas serum leptin and triggering receptor expressed on myeloid cells (TREM)-1 levels were positively correlated with BMI (**Table 3**). Serum IFN-alpha and IFN-gamma levels were significantly elevated in SLE patients with a BMI<20 compared to those with a BMI>20 (**Figures 2A, B**). Serum TREM-1 and leptin levels were significantly higher in SLE patients with a BMI>20 compared to those with a BMI<20 (**Figures 2C, D**). Taken together, BMI was associated with several serum cytokine levels in patients with SLE, especially higher serum IFN-alpha and IFN-gamma levels were observed in those with low body weight. Additionally, the association between serum cytokine levels and SLEDAI-2K scores was analyzed in patients with SLE (**Supplementary Table 2**). SLEDAI-2K scores significantly correlated with serum chemokines (CCL2, CX3CL1, and CXCL10), G-CSF, and IFN-alpha levels. Notably, these chemokines are induced in response to IFN stimulation (35). In the multiple regression analysis with SLEDAI-2K as the dependent variable and BMI and IFN-alpha as covariates, while the results did not reach statistical significance, BMI exhibited a tendency toward a greater absolute t-value compared to IFN-alpha (**Supplementary Table 3**). Importantly, causal mediation analysis revealed that BMI had a direct effect on SLEDAI-2K scores (beta -0.8866 (95%CI, -1.539, -0.002, *p* = 0.048), whereas no significant indirect effect was observed via IFN-alpha (beta -0.0635, 95%CI, -0.5213, 0.05, *p* = 0.150) (**Figure 2E**). This result suggested that other unknown BMI-related factors besides the IFN system may influence SLE disease activity.

**Table 3:** The correlation between BMI and serum cytokines.

Variable	Spearman Correlation	P-value
TNFα	0.0957877863	0.589966783
IL6	-0.1205565355	0.497043312
C5a	0.0009171508	0.995892651
S100A8	0.1260215588	0.477593084
IL10	-0.2830759098	0.104752055
CCL2	-0.2595536806	0.138240390
VEGF	0.2731580844	0.118031457
IL6Rα	0.0921736569	0.604135406
IL1β	-0.0659654900	0.710891900
IFNγ	-0.4282039280	0.011521852*
IL1ra	-0.1334454436	0.451820252
CCL3	-0.0335658734	0.850521481
CCL4	0.1988675963	0.259522299
IL17A	-0.0545406593	0.759333157
CX3CL1	-0.2425655595	0.166901596
M-CSF	-0.1343477617	0.448739689
G-CSF	0.0266921613	0.880886447
IFNα	-0.4526857116	0.007183051**
TREM-1	0.4164182831	0.014296904
GM-CSF	-0.2922936102	0.093452824
IL12/IL23p40	-0.3388257991	0.049972856*
IL18	-0.2421278151	0.167692354
Ghrelin	0.1250203444	0.526159909
Leptin	0.4201014267	0.026031116*
CXCL10	-0.3089292547	0.124634926

Correlations between serum cytokine levels and BMI analyzed by Spearman’s rank correlation coefficients in patients with SLE (n=34). *P < 0.05, **P <0.01. BMI, body mass index; TNF, tumor necrosis factor; IL, interleukin; IFN, interferon; CCL, C-C motif chemokine ligand; M-CSF, macrophage-colony stimulating factor; GM-CSF, granulocyte macrophage-colony stimulating factor; CX3CL, C-X3-C motif chemokine ligand.

### 3.4 BMI-related phenotypic and transcriptomic alterations in patients with SLE

**Figure 3:**
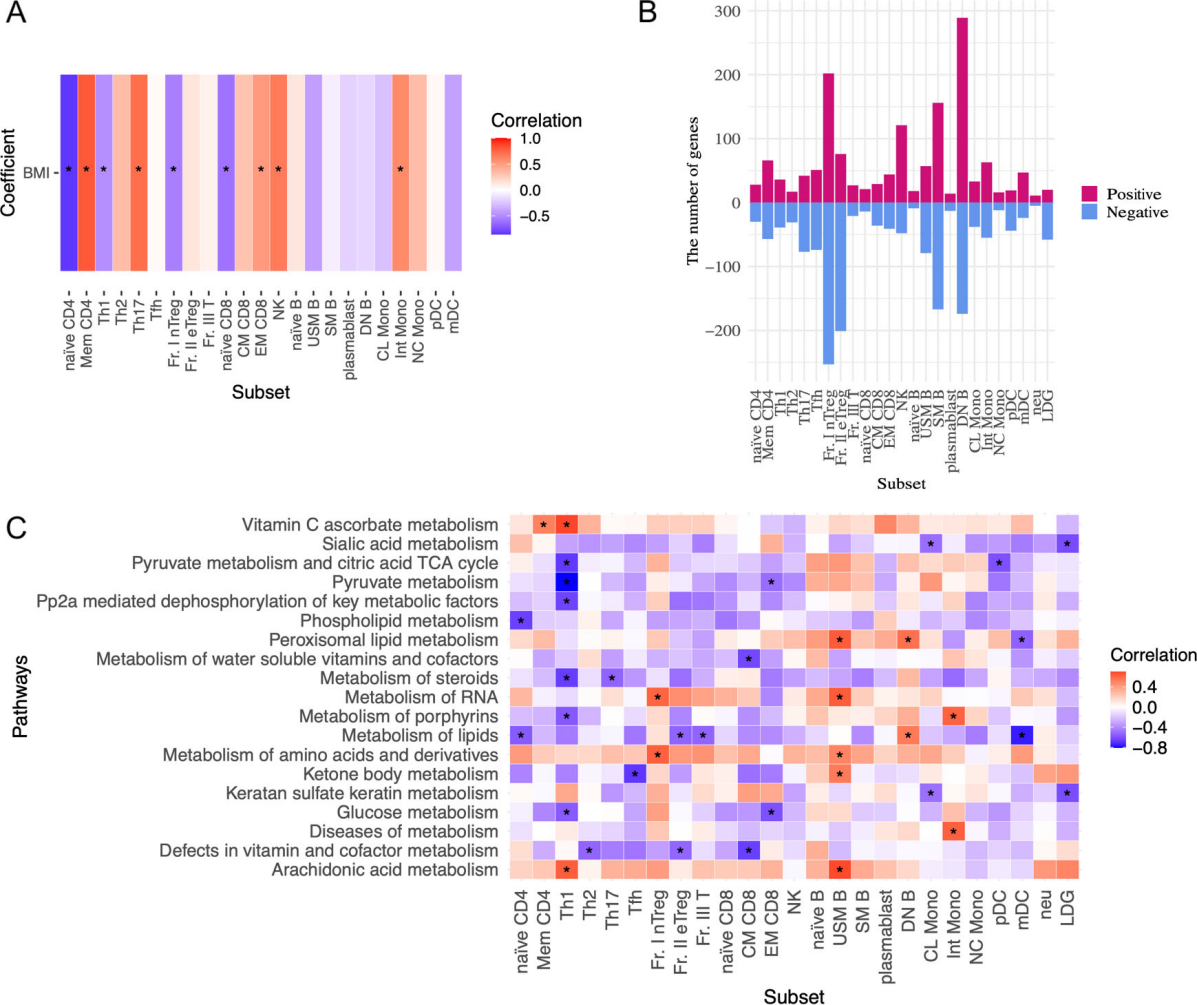
BMI-related immunological phenotypes and transcriptome analysis in patients with SLE. **(A)** Association between the frequencies of peripheral immune cell subsets and BMI analyzed by Spearman’s rank correlation coefficients. **(B)** The numbers of BMI-related gene expressions in each subset. Genes related to BMI were identified by regressing the expression levels of each subset gene against BMI, age, daily dosage of PSL, and library size as explanatory variables by the MASS package. A false discovery rate (FDR) of < 0.01 was regarded as significant. **(C)** Correlation between BMI and cell metabolism pathway and interferon signature scores analyzed by GSVA. Spearman’s rank correlation coefficients. Data with correlation coefficient >0.6 and p <0.05 are indicated. *P < 0.05. Abbreviations of cell subsets are listed in the Method section and the previous study (23, 31). BMI, body mass index; GSVA, gene-set variation analysis.

Next, we analyzed the association between the immunophenotyping of peripheral blood cells and BMI in patients with SLE (**Figure 3A**). BMI positively correlated with the frequencies of memory CD4^+^ T, Th17, effector memory (EM) CD8^+^ T, and NK cells. Whereas, BMI was negatively correlated with the frequencies of naïve CD4^+^ T, Th1, Fr. I nTreg, and naïve CD8^+^ T cells. Thus, BMI primarily influenced the altered frequencies of T cell subsets. Finally, we investigated BMI-related gene expression in the peripheral immune cells of patients with SLE on the ImmuNexUT database using the MASS package. Genes related to BMI were identified by regressing the gene expression levels of each subset against BMI, age, daily PSL dosage, and library size as explanatory variables. The numbers of these genes in each subset are shown in **Figure 3B**. BMI-related genes were enriched in cell subsets associated with the acquired immune system, including DNB, SM B, Fr. I nTreg, and Fr. II eTreg cells (**Figure 3B**). Pathway analysis of these BMI-related genes revealed subset-specific pathways, predominantly cellular metabolism-related pathways (**Figure 3C**, **Supplementary Figure 1**). For example, pathways involving BMI-negatively related genes included glucose metabolism-related pathways in Th1 and EMCD8 cells. In both subsets, Fr. I nTreg and Fr. II eTreg cells (**Supplementary Figure 1**), BMI was negatively correlated with phosphatidylinositol (PI)-related pathways. Importantly, these BMI-related pathways were primarily observed in cell subsets associated with the acquired immune system (**Figure 3C**, **Supplementary Figure 1**). Notably, the IFN signaling pathway was not associated with BMI in any cell subset, which aligned with the results of the mediation analysis (**Figure 2E**). Taken together, the ImmuNexUT database revealed that BMI-related phenotypic and transcriptomic alterations were predominantly observed in cell subsets related to the acquired immune system, especially in T cells, independently of IFN signaling.

## 4 Discussion

There have been numerous reports on the association between autoimmune diseases and patient physique; however, the significance of BMI in SLE remains unclear. One report from the Southern California Lupus Registry (SCOLR) (n=157) indicated a significant association between obesity and SLEDAI on multivariate analysis (odds ratio = 2.335, p=0.026) (21). Additionally, a recent study summarized the impact of obesity on disease activity in SLE (36). In contrast, another report from the Hopkins lupus cohort (n=2452) demonstrated higher SLEDAI scores in patients with cachexia (20). The researchers indicated that more than half of patients with SLE exhibited cachexia, and intermittent cachexia was associated with higher damage index scores. They suggested that cachexia is an under-recognized syndrome of SLE. We speculated that this discrepancy could be due to differences in the patient backgrounds of the cohorts. For example, glucocorticoid intake has effects on body weight gain and the frequency of current glucocorticoid users may have influenced the results. In the SCOLR, obese patients showed higher frequencies and doses of glucocorticoid than non-obese patients (46.0% vs 27.6%, p=0.029) (21). Although this hypothesis requires further evaluation in different cohorts, our study supported the association between a low BMI and diseases activity in SLE. Additionally, the SLE patients with a low BMI showed elevated serum IFN-alpha levels. It is well documented that IFN-alpha induces body weight loss and cachexia. For example, therapeutic administration of IFN-alpha for viral hepatitis frequently leads to body weight loss and appetite reduction (37). We speculated that excess secretion of IFN-alpha due to disease exacerbation could trigger cachexia as one of the systemic syndromes of lupus. Importantly, we demonstrated that low BMI was one of the independent explanatory variables for SLEDAI-2K, suggesting that cachexia is an important symptom of active SLE. Furthermore, constitutional and mucocutaneous symptoms, which are closely related to IFN (38, 39), were more frequently observed in SLE patients with a low BMI in our study. Recently, the anti-IFN RA antibody has emerged as a treatment option for SLE, and many studies have focused on identifying patients who would benefit the most from anti-IFN therapy. Our study suggested that SLE patients with a low BMI could be good candidates for anti-IFN therapy as a form of personalized medicine.

We firstly analyzed the BMI-related phenotypic and transcriptomic alterations in patients with active SLE. These alterations were primarily observed in cells related to the acquired immune system, such as T cells and B cells. Especially, the frequencies and glucose metabolism-related pathways in Th1 and EMCD8^+^ T cells were negatively associated with BMI. BMI had a direct effect on SLEDAI-2K scores, therefore, we speculated that other mechanisms of BMI-related immune alterations exist, independent of IFN signaling. Our previous study demonstrated that Th1 and EMCD8 T cells exhibited significantly greater gene expression variance in the patients with high disease activity and mucocutaneous lesions (24). These observations suggest that cachexia-induced metabolic alterations in Th1 and EMCD8^+^ T cells could play a role in the pathogenesis of SLE patients with a low BMI. Furthermore, previous studies have highlighted the immune-metabolic crosstalk, especially in cancer and infection. During cachexia, several types of immune cells, including M2 macrophages and CD8^+^ T cells, are activated (40). Baazim H, et al. showed that CD8^+^ T cell activation and expansion trigger the body weight loss through muscle atrophy. Notably, CD8^+^ T cell deficiency protects against cachexia in a viral infection model (41). These findings were consistent with our results. Regarding Treg cells, our data suggested that PI-related pathways were negatively associated with BMI. The activation of PI/Akt signaling inhibits Foxo transcription and impairs the development and function of Treg cells (42), therefore, Treg cells in patients with low BMI may be compromised. Treg cells not only regulate autoimmune responses in SLE (43), but also play a protective role against muscle catabolism (40). Thus, BMI-related phenotypic and transcriptomic alterations in cells related to the acquired immune response could contribute to cachexia as well as the pathogenesis of active SLE, independent of IFN signaling. Further studies are required to confirm these findings.

This study had some limitations. First, the number of patients with SLE was relatively small, which may have influenced the results. However, this study was the first to analyze the transcriptomic characteristics of patients with SLE in terms of body weight, and we believed that the transcriptome data from various immune cells provided valuable insights. Second, all the analyzed patients were of Asian descent, and the mean BMI was relatively low. Further studies are required to evaluate the effects of BMI on the pathogenesis of SLE in other populations. Third, the detailed mechanisms by which the physique induces phenotypic and transcriptomic alterations remain unclear. We suspected that the alterations in cell metabolism may be related, and our pathway analysis yielded consistent results. A fourth limitation of this study is the lack of consideration for cumulative steroid intake. Some of the recruited patients had received treatment at other hospitals at the onset of the disease, making it impossible to obtain accurate information on the cumulative steroid intake for all participants. Although this is beyond the scope of this study, further studies are required to address this issue.

In conclusion, we described the transcriptomic, immunological, and clinical characteristics associated with BMI in patients with SLE. Our analysis highlighted several distinct immunological features of active SLE patients with a low BMI. We found that serum levels of IFN-alpha were significantly elevated in SLE patients with a lower BMI, suggesting that low BMI, or cachexia, could be an explanatory factor for disease activity. Additionally, we revealed phenotypic and transcriptomic alterations, particularly in the cell subsets of the acquired immune system. These alterations in immune cells could contribute to both disease exacerbation and cachexia in SLE patients with a low BMI. Our findings emphasize the close association between physique, the acquired immune system, and autoimmune diseases, providing promising insights for personalized medicine in SLE.

## Data Availability

The datasets presented in this study can be found in online repositories. The names of the repository/repositories and accession number(s) can be found below: http://humandbs.biosciencedbc.jp/en, E-GEAD-397.

## Supplementary material

Supplementary Table 1 Clinical information. Clinical information of patients with SLE. Data are presented as the number (percentage) of patients, unless otherwise indicated. SD, standard deviation.

Supplementary Table 2 Correlation between serum cytokine levels and SLEDAI-2K. The Correlation between serum cytokine levels and the SLEDAI-2K components analyzed by Spearman’s rank correlation coefficients in patients with SLE (n=34). *P < 0.05, **P <0.01, ***P <0.001. SLEDAI, systemic lupus erythematosus disease activity index; BMI, body mass index; CRP, C reactive protein; ESR, erythrocyte sedimentation rate; ds-DNA Ab, double-stranded DNA antibody.

Supplementary Figure 1 The BMI-related pathways in patients with SLE. Correlation between BMI, and cell metabolism pathways and interferon signature scores analyzed by GSVA. Spearman’s rank correlation coefficients. *P < 0.05.
