# Supplementary material for: Body weight in systemic lupus erythematosus is associated with disease activity and the adaptive immune system, independent of type I IFN
Source: Front Immunol. 2025 Feb 18;16:1503559. doi: 10.3389/fimmu.2025.1503559 (PMC11876045; doi:10.3389/fimmu.2025.1503559)
Supplement: Supplementary Table 1 — Clinical information. Clinical information of patients with SLE. Data are presented as the number (percentage) of patients, unless otherwise indicated. SD, standard deviation. [file DataSheet1.zip › Table 3.docx]

Supplementary Table 3. The multiple regression analysis

| Indicators | Estimate | Std.error | t-value | p-value |
| --- | --- | --- | --- | --- |
| (Intercept) | 30.74873 | 10.13754 | 3.033 | 0.00486** |
| BMI | -0.88659 | 0.45928 | -1.930 | 0.06275 |
| IFN alpha | 0.02096 | 0.03902 | 0.537 | 0.59493 |
