# Supplementary material for: Body weight in systemic lupus erythematosus is associated with disease activity and the adaptive immune system, independent of type I IFN
Source: Front Immunol. 2025 Feb 18;16:1503559. doi: 10.3389/fimmu.2025.1503559 (PMC11876045; doi:10.3389/fimmu.2025.1503559)
Supplement: Supplementary Table 1 — Clinical information. Clinical information of patients with SLE. Data are presented as the number (percentage) of patients, unless otherwise indicated. SD, standard deviation. [file DataSheet1.zip › Table 2.docx]

# Supplement Table 2. The correlation between SLEDAI2K score and serum cytokines

| **Variable** | **Spearman Correlation** | **P-value** |
| --- | --- | --- |
| TNFα | 0.2817201 | 0.10649753 |
| IL6 | 0.1922317 | 0.27608119 |
| C5a | -0.2120268 | 0.22865919 |
| S100A8 | -0.1832373 | 0.29959679 |
| IL10 | 0.2645319 | 0.13057043 |
| CCL2 | 0.3950991 | 0.02074350* |
| VEGF | -0.1153585 | 0.51590905 |
| IL6Rα | 0.1741868 | 0.32450483 |
| IL1β | 0.2189346 | 0.21349761 |
| IFNγ | 0.1905566 | 0.28036709 |
| IL1ra | 0.3200318 | 0.06501499 |
| CCL3 | 0.1196621 | 0.50026459 |
| CCL4 | 0.1117333 | 0.52927261 |
| IL17A | 0.2737120 | 0.11725825 |
| CX3CL1 | 0.4216991 | 0.01299102* |
| M-CSF | 0.2328233 | 0.18513871 |
| G-CSF | 0.3450873 | 0.04563014* |
| IFNα | 0.3810158 | 0.02620234* |
| TREM-1 | -0.0671061 | 0.70611271 |
| GM-CSF | 0.3110000 | 0.07340751 |
| IL12/IL23p40 | 0.2789265 | 0.11016315 |
| IL18 | 0.1551901 | 0.38082043 |
| Ghrelin | -0.1056284 | 0.59268425 |
| Leptin | -0.2944172 | 0.12830553 |
| CXCL10 | 0.4329063 | 0.02717348* |
