# Supplementary material for: Body weight in systemic lupus erythematosus is associated with disease activity and the adaptive immune system, independent of type I IFN
Source: Front Immunol. 2025 Feb 18;16:1503559. doi: 10.3389/fimmu.2025.1503559 (PMC11876045; doi:10.3389/fimmu.2025.1503559)
Supplement: Supplementary Table 1 — Clinical information. Clinical information of patients with SLE. Data are presented as the number (percentage) of patients, unless otherwise indicated. SD, standard deviation. [file DataSheet1.zip › Table 1.docx]

Supplement Table 1. The clinical information of Serum cohort

| **Variable** | **n = 34** |
| --- | --- |
| **Age (year), median (SD)** | 48.6 (14.6) |
| **Female Sex** | 28 (82%) |
| **BMI, median (SD)** | 21.6 (2.9) |
| **Prednisolone (mg), median (SD)** | 6.3 (11.9) |
| **Hydroxychloroquine (mg)** | 7 (21%) |
| **Mycophenolate mofetil (mg)** | 3 (8.8%) |
| **Calcineurin inhibitor (mg)** | 5 (15%) |
| **Azathioprine (mg)** | 2 (5.9%) |
| **Belimumab** | 0 (0%) |
| **SLEDAI2K** | 11.8 (7.6) |
| **Seizure** | 0 (0%) |
| **Psychosis** | 1 (2.9%) |
| **Organic Brain Syndrome** | 0 (0%) |
| **Visual Disturbance** | 5 (14.7%) |
| **Cranial Nerve Disorder** | 1 (2.9%) |
| **Lupus headache** | 2 (5.9%) |
| **Cerebrovascular accident** | 0 (0%) |
| **Vasculitis** | 1 (2.9%) |
| **Arthritis** | 14 (41.1%) |
| **Myositis** | 3 (8.8%) |
| **Urinary casts** | 9 (26.4%) |
| **Hematuria** | 5 (14.7%) |
| **Proteinuria** | 8 (23.5%) |
| **Pyuria** | 5 (14.7%) |
| **New Rash** | 6 (17.6%) |
| **Alopecia** | 4 (11.7%) |
| **Mucosal ulcers** | 3 (8.8%) |
| **Pleurisy** | 6 (17.6%) |
| **Pericarditis** | 4 (11.7%) |
| **Low complement** | 25 (73.5%) |
| **Increased anti-DNA antibodies** | 16 (47.0%) |
| **Fever** | 5 (14.7%) |
| **Thrombocytopenia** | 4 (11.7%) |
| **Leukopenia** | 8 (23.5%) |
| Data were presented as number (percentage) of patient unless otherwise indicated. | |

Abbreviation: SD, standard deviation
