# Supplementary figures and images for: Body weight in systemic lupus erythematosus is associated with disease activity and the adaptive immune system, independent of type I IFN
Source: Front Immunol. 2025 Feb 18;16:1503559. doi: 10.3389/fimmu.2025.1503559 (PMC11876045; doi:10.3389/fimmu.2025.1503559)

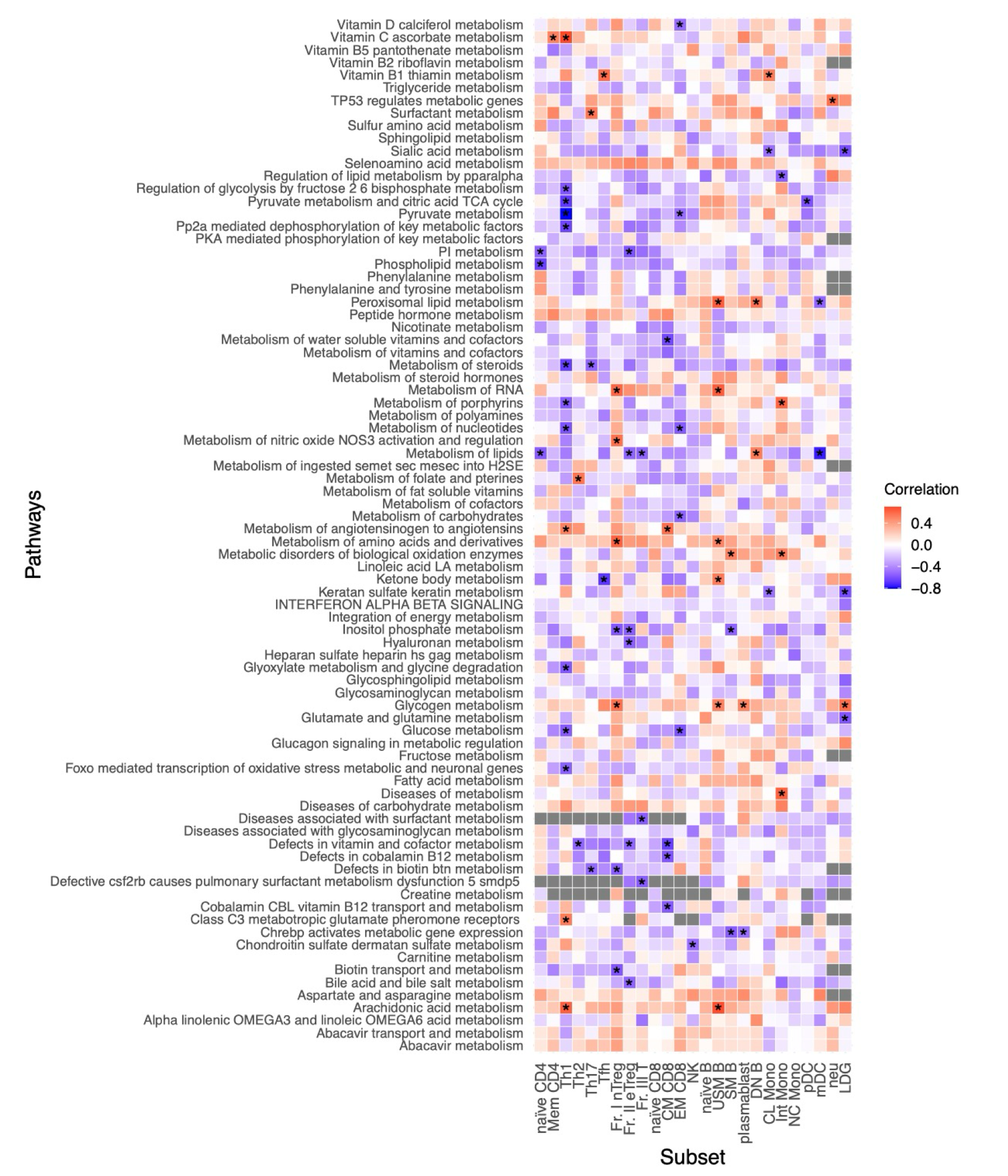

Supplement: Supplementary Table 1 — Clinical information. Clinical information of patients with SLE. Data are presented as the number (percentage) of patients, unless otherwise indicated. SD, standard deviation. [file DataSheet1.zip › Image 1.TIFF]
